# Pulsatile Tinnitus: Differential Diagnosis and Radiological Work-Up

**DOI:** 10.1007/s40134-017-0199-7

**Published:** 2017-01-24

**Authors:** Sjoert A. H. Pegge, Stefan C. A. Steens, Henricus P. M. Kunst, Frederick J. A. Meijer

**Affiliations:** 1 Department of Radiology and Nuclear Medicine, Radboud University Medical Center Nijmegen, Geert Grooteplein 10, P/O Box 9101, 6500 HB Nijmegen, The Netherlands; 2 Department of Otorhinolaryngology, Radboud University Medical Center Nijmegen, P/O Box 9101, Nijmegen, The Netherlands

**Keywords:** Pulsatile Tinnitus, Multi-detector CT, MRI, Angiography

## Abstract

**Purpose of Review:**

Identification of the underlying cause of pulsatile tinnitus is important for treatment decision making and for prognosis estimation. For this, an adequate diagnostic imaging strategy is crucial.

**Recent Findings:**

Both CT and MRI can be useful, and in general, these modalities provide complementary diagnostic information. The scanning protocol can be optimized based on the estimated a priori chance for finding specific pathology, or the need to rule out more rare but clinical significant disease. In recent years, dynamic CTA, also referred to as 4D-CTA, has become available as a new technique that enables non-invasive evaluation of hemodynamics for the detection, classification, and follow-up of vascular malformations.

**Summary:**

The value of different diagnostic imaging modalities in the work-up of pulsatile tinnitus is discussed in relation to the differential diagnosis. Furthermore, imaging findings of different diseases are presented, both for CT and MRI.

**Electronic supplementary material:**

The online version of this article (doi:10.1007/s40134-017-0199-7) contains supplementary material, which is available to authorized users.

## Introduction

Tinnitus is defined as an auditory perception of internal origin, and can have a significant influence on the well-being and performance in daily activities of affected subjects [1]. The auditory perception differs between patients and is described diversely, such as a buzzing, ringing, or whistling tone, and can be perceived as either pulsatile or non-pulsatile. In pulsatile tinnitus, the auditory perception is repetitively synchronous to the patient’s heartbeat. All other auditory perceptions are considered non-pulsatile. Less than 10% of patients presenting with tinnitus have pulsatile tinnitus [2].

In about 70% of the cases with pulsatile tinnitus, an underlying cause can be identified by adequate diagnostic work-up [3]. Vascular causes include arterial or venous vascular pathologies, such as dural arteriovenous fistula (dAVF), arteriovenous malformation (AVM), aneurysm, internal carotid artery stenosis or dissection, congenital vascular variants, transverse sinus stenosis, or increased cardiac output [4, 5]. Non-vascular etiologies of pulsatile tinnitus include neoplasm like paraganglioma, osseous pathology, idiopathic intracranial hypertension, and systemic disorders such as anemia [6–8]. Pulsatile tinnitus is perceived unilaterally in most cases, though it can occur bilaterally in case of systemic vascular disease or the presence of a midline vascular lesion, e.g., superior sagittal sinus AVF. Bilateral pulsatile tinnitus without a vascular cause has also been described in somatosensory pulsatile tinnitus [9]. This is a form of tinnitus that can be aroused or changed by stimulation of the cerebral somatosensory, somatomotor, and visual-motor circuits, e.g., by pressure on myofacial trigger points, specific eye-movements, or powerful muscle contractions.

When the auditory perception is only perceived by the patient and cannot be heard by the clinician by auscultation, it is called subjective tinnitus. In case of objective tinnitus, which can be heard by the clinician, more frequently the etiology can be found by ancillary investigations and there is usually a genuine physical source of sound in contrary to subjective tinnitus [10]. Subjective tinnitus is more prevalent than objective tinnitus. The etiology of subjective tinnitus often lies in otologic disorders that also lead to conductive or sensorineural hearing loss [10, 11]. Conductive hearing loss may be caused by impaction of cerumen, external or internal otitis, cholesteatoma, ossicular chain abnormalities, or tympanic membrane perforation [12]. Sensorineural hearing loss is caused by disease or abnormality at the level of the inner ear or eighth cranial nerve, and etiologies include noise-induced hearing loss, Meniere’s disease, or acoustic neurinoma [13–15].

Identification of the underlying cause of pulsatile tinnitus is important for adequate treatment and for prognosis estimation. Different guidelines are available for the diagnostic work-up of pulsatile tinnitus [3, 10, 11, 16]. Complete and detailed history taking is essential, which includes questioning for possible accompanying complaints like vertigo, hearing loss, otorrhoea, and otalgia as well as the course of symptoms. In addition, one needs to be aware of possible accompanying neurological deficits. Next, an otologic physical examination needs to be performed. Using otoscopy, one can already evaluate the presence of a tympanic cavity mass. An underlying vascular etiology can be suspected when pulsatile tinnitus is influenced by vascular compression or when a vascular bruit is heard by auscultation. Audiometric evaluation may also reveal a possible cause of tinnitus, such as noise-induced hearing loss or otosclerosis.

In this review, we will discuss imaging strategies for the diagnostic work-up of pulsatile tinnitus. Furthermore, the differential diagnosis of pulsatile tinnitus is discussed and imaging findings of different diseases are presented, both for CT and MRI. We differentiate vascular, neoplastic, and osseous etiologies.

### Radiological Work-Up: What Imaging Strategy to Choose?

Considering the broad differential diagnosis of pulsatile tinnitus, the optimal diagnostic imaging strategy depends on the initial clinical evaluation. Both CT and MRI can be useful, and in general, these modalities are complementary. The scanning protocol can be optimized based on the estimated a priori chance for finding specific pathology, or the need to rule out more rare but clinical significant disease. The value of imaging modalities for the detection and characterization of different pathologies, which can cause pulsatile tinnitus, is presented in Table 1.

**Table 1:** Value of imaging modalities for the detection and characterization of different pathologies which can cause pulsatile tinnitus

Pathology	Non-enhanced CT	Conventional CTA/CTV^a^	4D-CTA^a^	Conventional MRI study	MRA/MRV	DSA	Duplex ultrasound
Tympanic cavity pathology	+++	+++	+++	–	–	–	–
Temporal bone pathology, e.g., otosclerosis, Paget disease, and LCH	+++	+++	+++	+	–	–	–
Paraganglioma	++	++	++	+++	+++	+	+
Hypervascular metastasis or meningeoma	+	++	+	+++	±	–	–
Vascular channel dehiscence or variant	+++	+++	+++	–	–	–	–
Aberrant ICA or stapedial artery	+++	+++	+++	–	±	+++	–
Vascular loops, neurovascular conflict	–	+	+	+++	++	–	–
Arteriovenous fistula	–	+	+++	±	++	+++	±
Arteriovenous malformation	–	++	++	++	++	+++	+ (superficial)
Vessel wall pathology, e.g., atherosclerosis, FMD, or dissection	–	++	+	±	+++	+	++
Idiopathic intracranial hypertension	–	–	–	+++	±	–	–

+++ Most optimal, ++ good, + moderate, ± indirect signs, – not suitable, *LCH* Langerhans cell histiocytosis, *ICA* internal carotid artery, *FMD* fibromuscular dysplasia

^a^Bone window CT reconstructions of the temporal bone can be obtained from CTA/CTV or 4D-CTA

For screening for underlying pathology and for the evaluation of a possible soft tissue mass or intracranial pathology, initial evaluation with MRI and MR angiography (MRA) is recommended with reported high diagnostic accuracy [17•]. An appropriate MRI protocol for the evaluation of cochlear and retro-cochlear pathology includes at least T1-W and 3D T2-W sequences of the skull base and the posterior fossa. Intravenous administration of a contrast agent, gadolinium, should be considered for the detection of possible labyrinth or cranial nerve enhancement, and for the detection of a soft tissue mass. A subsequent contrast-enhanced or time of flight MRA is advised, in case a paraganglioma or vascular malformation is suspected. The inclusion of a sequence covering the whole head, a T1-W, T2-W, or FLAIR sequence, needs to be considered for the evaluation of intracranial space-occupying or vascular pathology.

For the evaluation of osseous pathology of the temporal bone, a limited scanning range of thin-sliced (submillimetric) CT is sufficient. Multiplanar reconstruction (MPR) of the acquisition is crucial for adequate evaluation, such as the identification of bony dehiscence of vascular canals or the skull base.

Multi-detector CTA or CT venography (CTV) of the head and neck region can be performed for the evaluation of vascular pathology. Bone window images of the skull and temporal bone can be reconstructed from a multi-detector CTA or CTV acquisition, obviating the need for a separate acquisition, which reduces radiation exposure. While the anatomic evaluation of multi-detector CTA/CTV is excellent, the evaluation of flow dynamics is limited because only a single time point is obtained during the passage of a contrast bolus. Dynamic CTA, also referred to as 4D-CTA, is a technique that combines the non-invasive nature of CTA with the dynamic acquisition of digital subtraction angiography (DSA) [18•]. 4D-CTA enables the evaluation of flow dynamics of vasculature by multiple subsequent CT acquisitions, or a continuous volume CT acquisition, for a period of time. The coverage and temporal resolution of 4D-CTA depend on the width of the CT detector. Detector configurations that cover the whole head with 16-cm coverage are available from 2 major vendors, either as 320 × 0.5 mm or 256 × 0.625 mm collimations [18•]. A temporal resolution up to 20 frames/sec can be achieved from a continuous volume acquisition. Scanners with 4- to 8-cm coverage acquire smaller portions of the vascular system. An advantage of 4D-CTA over dynamic MRA is that 4D-CTA is not limited by the trade-off between temporal and spatial resolution [19]. The radiation dose of 4D-CTA should however be kept as low as possible, which can be achieved by implementing adequate filtering and image registration techniques. 4D-CTA generates a large amount of data, which requires powerful workstations and optimized data processing. In case a vascular malformation such as a dAVF is considered as a possible cause of pulsatile tinnitus, 4D-CTA can be a non-invasive alternative to DSA.

The role of DSA in the diagnostic work-up of pulsatile tinnitus has been minimized, and should be reserved for the indication to rule out vascular pathology in case MRI/MRA and CT/(4D-)CTA have not revealed the cause of pulsatile tinnitus. DSA can also be performed for the purpose of treatment planning, e.g., for determining the exact angioarchitecture of a vascular malformation or in preoperative tumor embolization.

The role of duplex ultrasound in the diagnostic work-up of pulsatile tinnitus is limited, although duplex ultrasound is an effective screening tool for the evaluation of superficial tissue structures or superficial vascular malformations and for the evaluation of vessel wall pathology of the carotid arteries.

## Differential Diagnosis and Imaging Findings

### Vascular Etiologies

#### Arteriovenous Malformation and Dural Arteriovenous Fistula

An AVM consists of a network of tortuous dilated arteries and veins, referred to as a nidus, through which shunting occurs between arteries and veins without the presence of a normal intervening arteriole-capillary bed. Typically, an AVM develops in adolescence or young adulthood but can remain occult for a long period [20]. An AVM located in the head and neck region can be the cause of pulsatile tinnitus.

An AVF is an, usually acquired, abnormal connection between an artery and a vein without an intervening nidus. Located along the dura or within a dural sinus, these are called dural AVF (dAVF). A direct arteriovenous shunt between a cerebral artery and a cortical draining vein is called a pial AVF, and occur within the brain or along the brain surface. An AVF can have a simple (single AVF) or more complex (multihole AVF) angioarchitecture.

The pathophysiology of dAVFs is controversial, but mostly considered a result of local hypoperfusion within a thrombosed dural sinus [21]. Progressive angiogenesis is triggered in the dural sinus wall and proliferation of microvascular networks develops into a plexus of venous channels, leading to an AVF. Pulsatile tinnitus results from high-flow passage through the sigmoid and petrosal sinus.

AVF is more frequent in pulsatile tinnitus than AVM, with reported prevalence numbers varying from 2 to 27% [6, 22, 23].

**Fig. 1:**
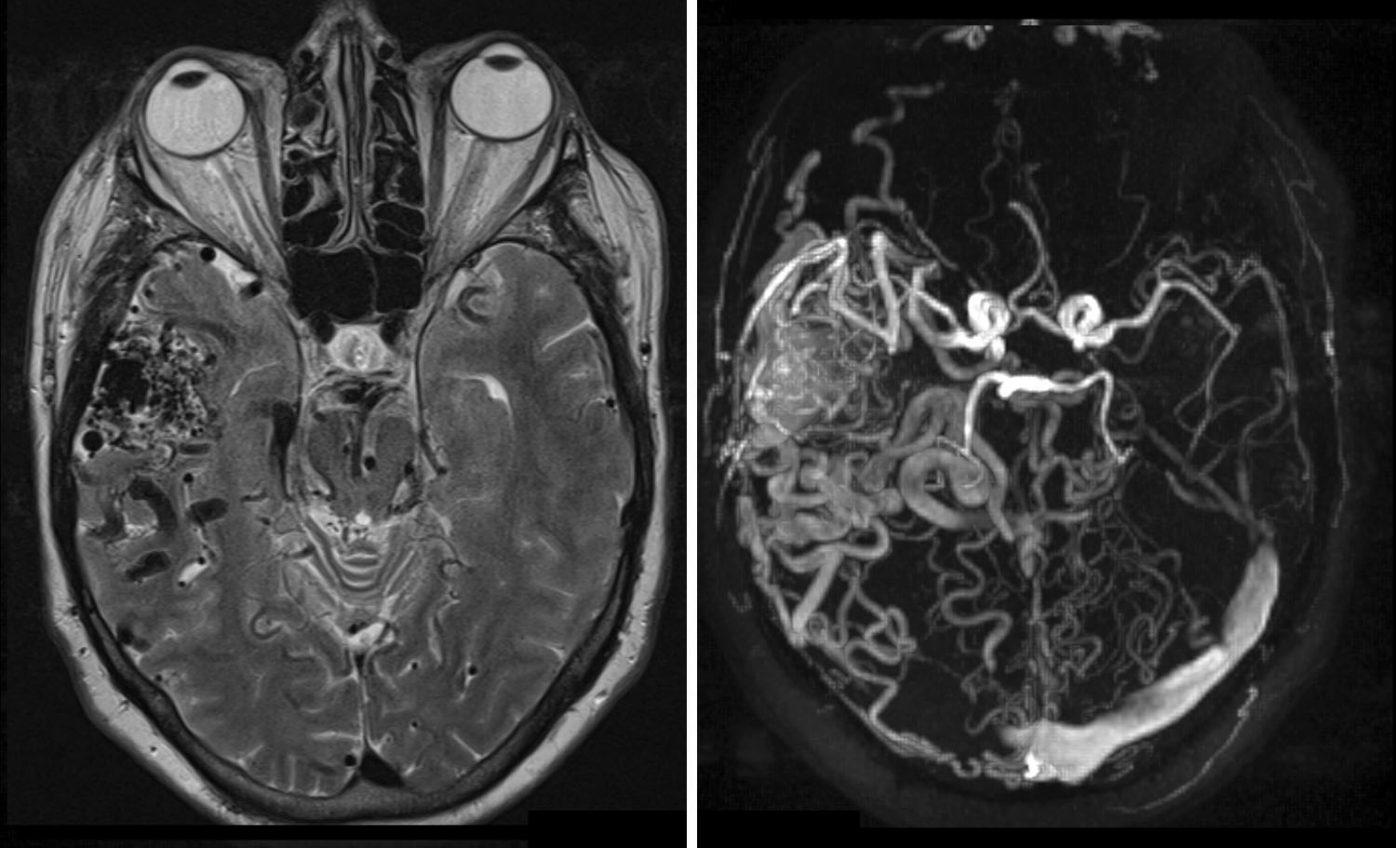
T2-W (*left*) and phase-contrast MRA (*right*) demonstrating intracranial arteriovenous malformation (AVM) located in the right temporal fossa

**Fig. 2:**
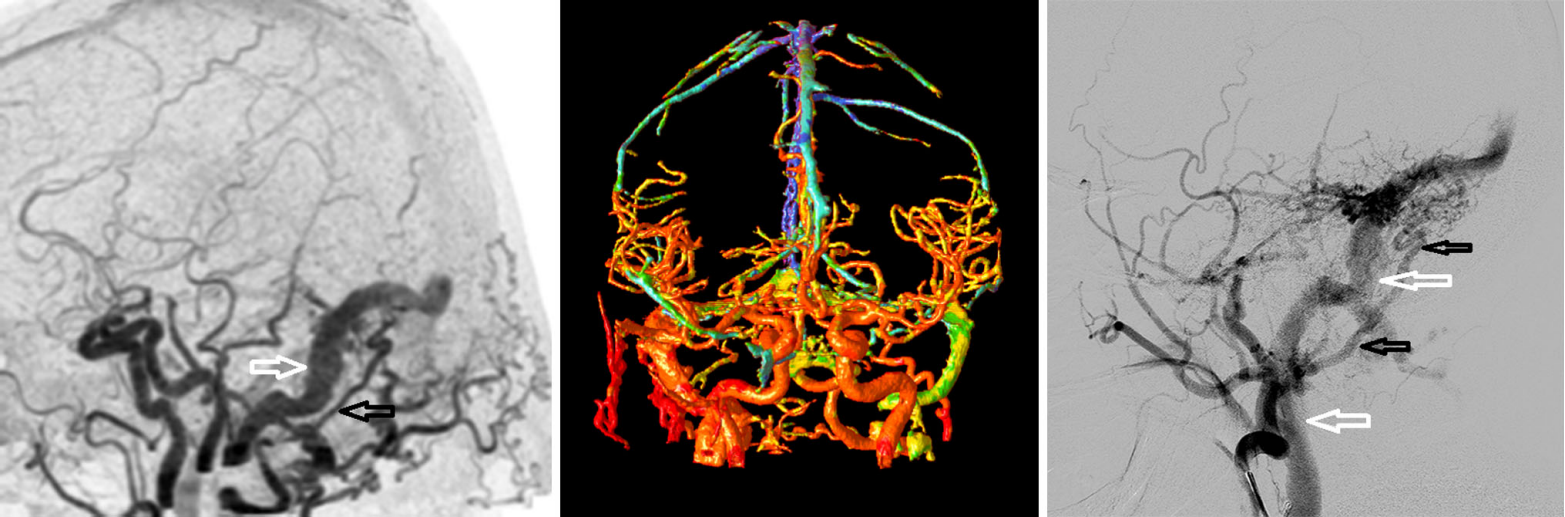
Dural arteriovenous fistula (dAVF) located in the right sigmoid sinus as identified by 4D-CTA and DSA. *Left* 4D-CTA lateral subtracted MIP demonstrating abnormal early contrast filling of the sigmoid sinus (*white arrow*) consistent with dAVF. Hypertrophic occipital artery identified as arterial feeder (*black arrow*). Anterograde venous drainage in the jugular vein. *Middle* Color-coded processing of 4D-CTA. Early contrast enhancement (*arterial flow*) is coded as *red-orange*, delayed contrast enhancement is coded as *yellow-green*. Notice the *red-colored*, hypertrophic occipital artery on the right side serving as arterial feeders of the dAVF. *Right* DSA, selective contrast injection of the external carotid artery showing a hypertrophic tortuous occipital artery (*black arrows*). Venous drainage of the sigmoid sinus into the jugular vein (*white arrows*) (Color figure online)

The nidus and tortuous vessels of an AVM can be detected either by MRI/MRA or CTA (Fig. 1), although the evaluation of the precise angioarchitecture of an AVM or micro-AVM is better depicted on DSA or 4D-CTA [24••, 25]. The detection of a dAVF is challenging on MRI/MRA and conventional CTA because frequently only indirect signs of a dAVF are visible, such as dilated vessels, cerebral edema, or (micro)hemorrhage. The evaluation of flow dynamics is limited on MRI/MRA and conventional CTA, and therefore DSA is considered the gold standard for the detection and evaluation of a dAVF. As DSA is a invasive procedure, it bears a small but non-neglectable risk of neurological complications [26]. There is increasing evidence that 4D-CTA has added value over conventional CTA for the diagnosis, treatment planning, and follow-up of dAVF [27••, 28]. Abnormal venous drainage is the hallmark for classifying and treatment decision making of a dAVF. The Borden and Cognard classifications are most commonly used for dAVFs [29, 30]. Retrograde venous flow in cortical veins, which is associated with increased risk of intracranial hemorrhage, can be adequately visualized by 4D-CTA [31]. An example of a multihole dAVF located in the sigmoid sinus as identified by 4D-CTA and DSA is provided in Fig. 2.

The main arterial feeders and the patterns of venous drainage of a dAVF or AVM as detected by 4D-CTA or MRA seem to be sufficient in most cases to correctly identify and classify an AVM or AVF, which could save the patient a pre-treatment invasive DSA [27, 28, 32]. In addition, 4D-CTA could replace invasive DSA in the follow-up of head and neck vascular malformations. The decrease in spatial resolution in comparison to DSA does not seem to change clinical management for most patients. Recently, carotid duplex ultrasound focusing on low resistance indexes of the external carotid and occipital arteries has been reported as a possible screening tool for dAVF in patients with pulsatile tinnitus [33]. The role of 4D-CTA and duplex ultrasound in the diagnostic work-up of tinnitus still need to be further addressed in larger cohort studies.

#### Vascular Stenoses

In the elderly population, atherosclerotic disease of the carotid or vertebral arteries is thought to be the most common cause of pulsatile tinnitus [34]. In a significant stenosed or occluded artery, increased vascular flow on the contralateral side could lead to pulsatile tinnitus as a symptom.

Fibromuscular dysplasia (FMD) is a segmental non-atheromatous, non-inflammatory vascular disease of unknown etiology. Often it is a disease of the young leading to vascular stenosis and cerebral ischemia. In medium-sized arteries, like the vertebral and carotid arteries, fibroblast-like changes of the smooth muscle cells cause narrowing of the arteries and seem to cause pulsitale tinnitus more frequently than in atherosclerotic disease. This is probably due to the location of arterial stenosis in FMD. In FMD, stenosis of the carotid artery is frequently located at the upper cervical level, resulting in easily transmitted vascular turbulence to the temporal bone. The classical imaging appearance of FMD is the so-called “string of beads” pattern shown on angiographic studies.

*Vascular loops and elongated arteries* are occasionally described as a possible cause of pulsatile tinnitus [35, 36]. Considering the presence of these vascular loops and elongations also in asymptomatic patients, other possible causes of pulsatile tinnitus need to be ruled out in those subjects.

#### Persistence of the Stapedial Artery

**Fig. 3:**
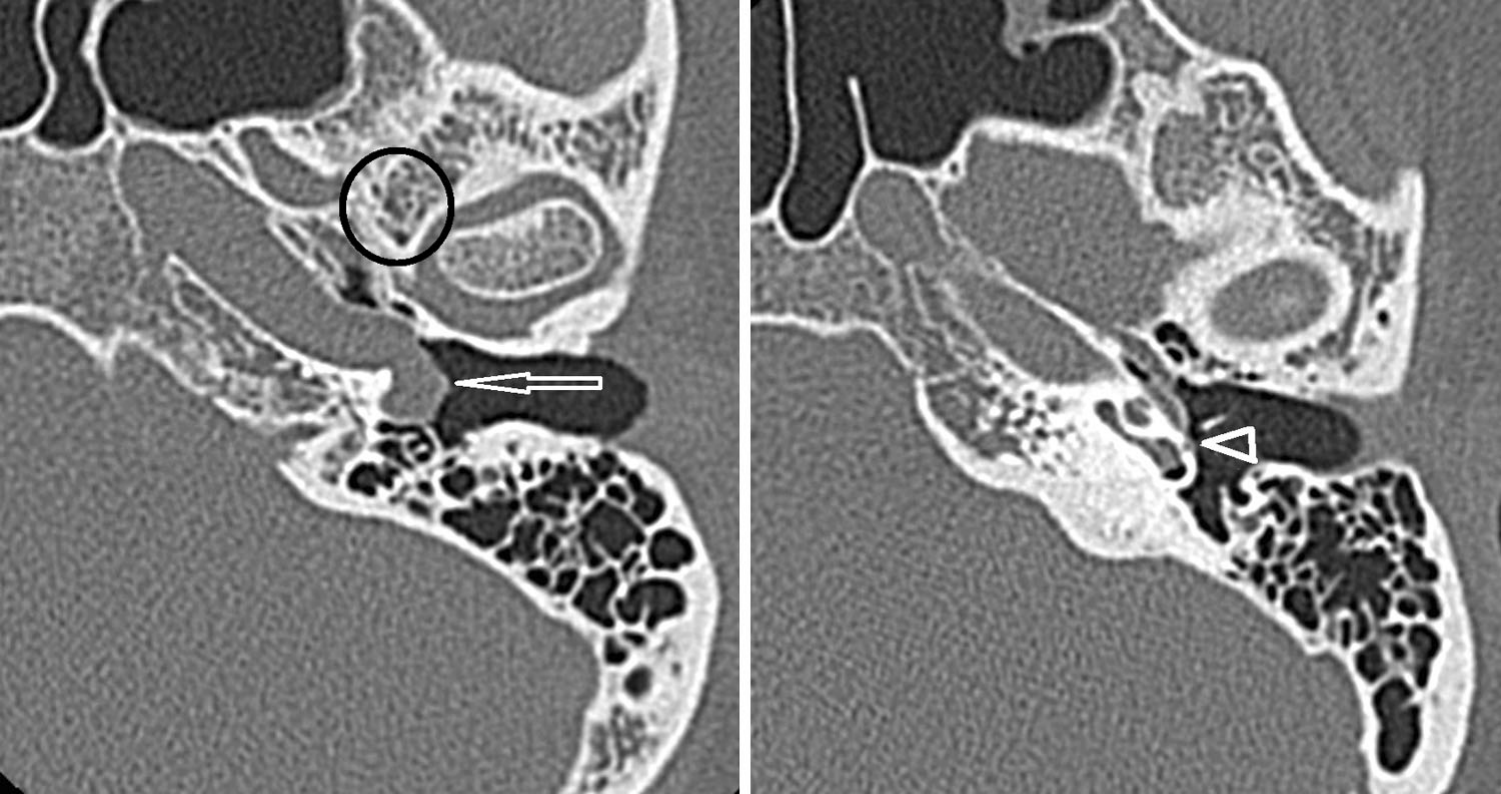
Aberrant course of the internal carotid artery (*arrow*) and persistence of the stapedial artery (*arrowhead*) on thin-sliced CT. Note the absence of the foramen spinosum (*encircled*)

An aberrant course of the internal carotid artery and persistence of the stapedial artery are congenital variants that need to be recognized on imaging studies. An aberrant course of the internal carotid artery in the middle ear may mimic a soft tissue mass or paraganglioma at otoscopy. The aberrant carotid artery enters the tympanic cavity via an enlarged tympanic canaliculus and then runs though the middle ear where it, due to a dehiscence in the carotid plate, enters the horizontal carotid canal (Fig. 3). The ascending carotid canal on the affected side has not developed and is therefore absent on CT or MRI.

A persistent stapedial artery fails to regress in early fetal development. As a result, the proximal course of the middle meningeal artery will not develop and the foramen spinosum will be absent (Fig. 3). In addition, CT may show subtle enlargement of the tympanic segment of the facial nerve canal in the coronal plane. These findings are therefore indirect signs for possible persistence of the stapedial artery, especially because the persistent stapedial artery itself is usually hardly visible on MRI/MRA. However, one should consider that in about 3% of the cases, the foramen spinosum is absent on CT [37]. Both CTA or DSA can be used for confirmation.

#### Venous Tinnitus

Venous tinnitus is heard as a continuous murmer that exaggerates in systole. By light pressure on the ipsilateral jugular vein, the murmur decreases. Light pressure on the contralateral jugular vein will increase the murmur. Rotating the patient’s head away from the involved side may relieve the murmur. Symptoms may increase by rotating the head toward the involved side. There seems to be an association with congenital variants such as a high-riding, enlarged, or diverticulum of the jugular bulb, which can be best depicted on thin-sliced high-resolution CT [38]. Prevalence of sigmoid sinus diverticulum and dehiscence has been reported to be significantly higher in pulsatile tinnitus than in the general population [39•].

### Paraganglioma

Paraganglioma, also known as glomus tumor, is the most frequent neoplastic cause of pulsatile tinnitus [40, 41]. Most paragangliomas are sporadic, about 7–10% are familial and usually autosomal dominant in inheritance [42]. In case of familial paragangliomas, they are frequently multicentric (35–50%) and can be associated with multiple endocrine neoplasia (MEN IIa and IIb) or phakomatoses [42].

Being a highly vascularized lesion, it is one of the most common causes of pulsatile tinnitus. Involving only the jugular bulb (glomus jugulare), the middle ear or mastoid (glomus tympanicum) or both (glomus jugulotympanicum), most of the paragangliomas located in the temporal bone will present with pulsatile tinnitus [41]. Bilateral pulsatile tinnitus is described in about 10% of cases due to a possible bilateral localization of paraganglioma [40, 43]. In contrary, pulsatile tinnitus is generally not a presenting symptom in a vagal paraganglioma (located along the vagal nerve) or carotid body paraganglioma (located at the carotid bifurcation).

A tympanic paraganglioma can be detected by otoscopic inspection as a red, pulsatile tympanic mass and can be very small at presentation measuring only a few millimeters. Paragangliomas grow along planes of least resistance following existing pathways, through bony canals or along the vessels and nerves. A tympanic paraganglioma can arise from glomus bodies anywhere along the Jacobsen nerve, a tympanic branch of the glossopharyngeal nerve.

**Fig. 4:**
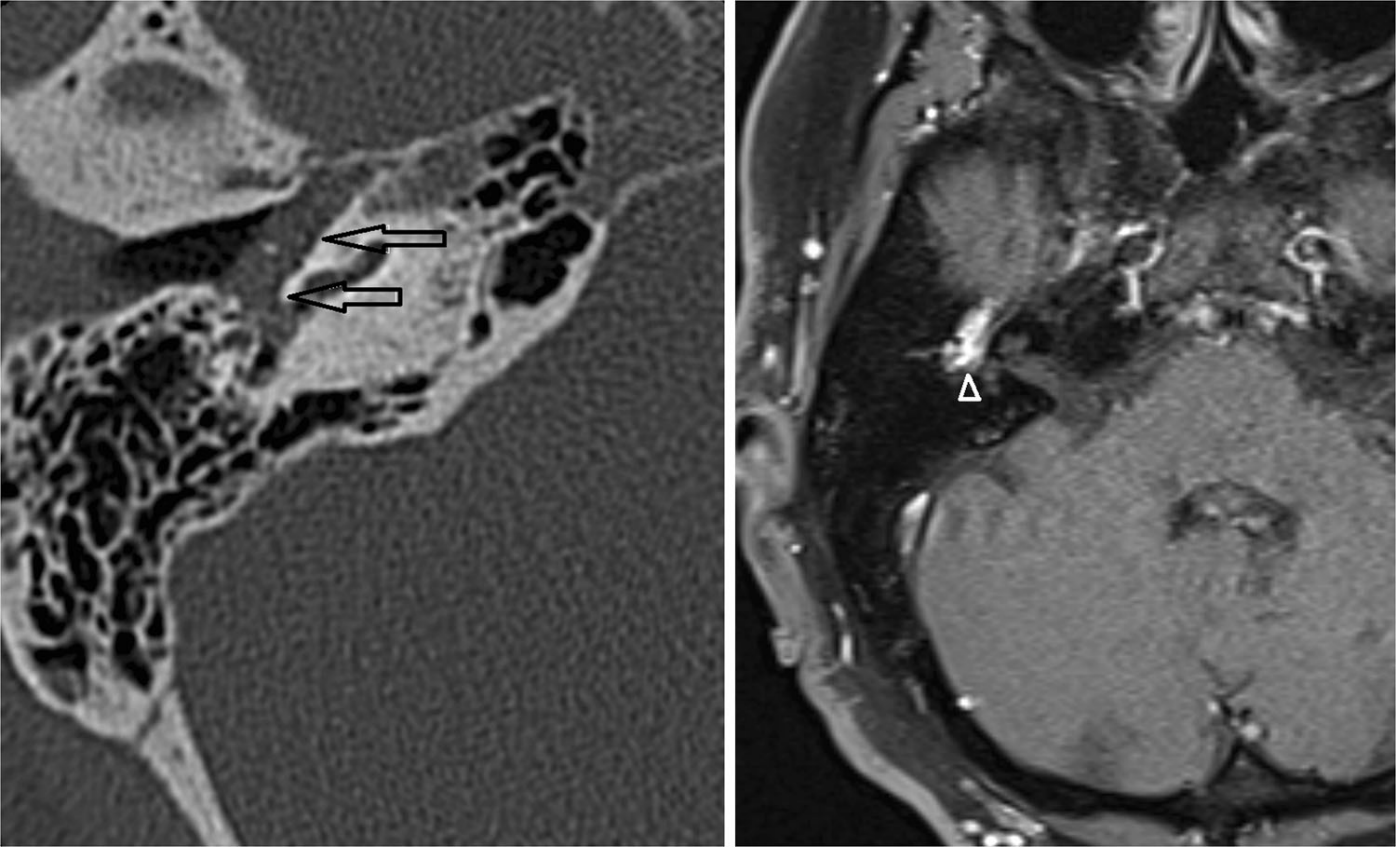
Glomus tympanicum. *Left* Axial CT shows a soft tissue mass in the middle ear (*arrows*). No visible bony erosion. *Right* Axial contrast-enhanced T1-W with fat suppression demonstrates strong enhancement of this lesion (*arrowhead*)

Both CT and MRI can be used for the detection and evaluation of a paraganglioma. The majority of tympanic paragangliomas are located on the promontory as a small well-defined tympanic mass with soft tissue mass. Usually, there is no or little surrounding bone erosion. These small tumors are best evaluated using thin-sliced CT with a bone algorithm (Fig. 4).

**Fig. 5:**
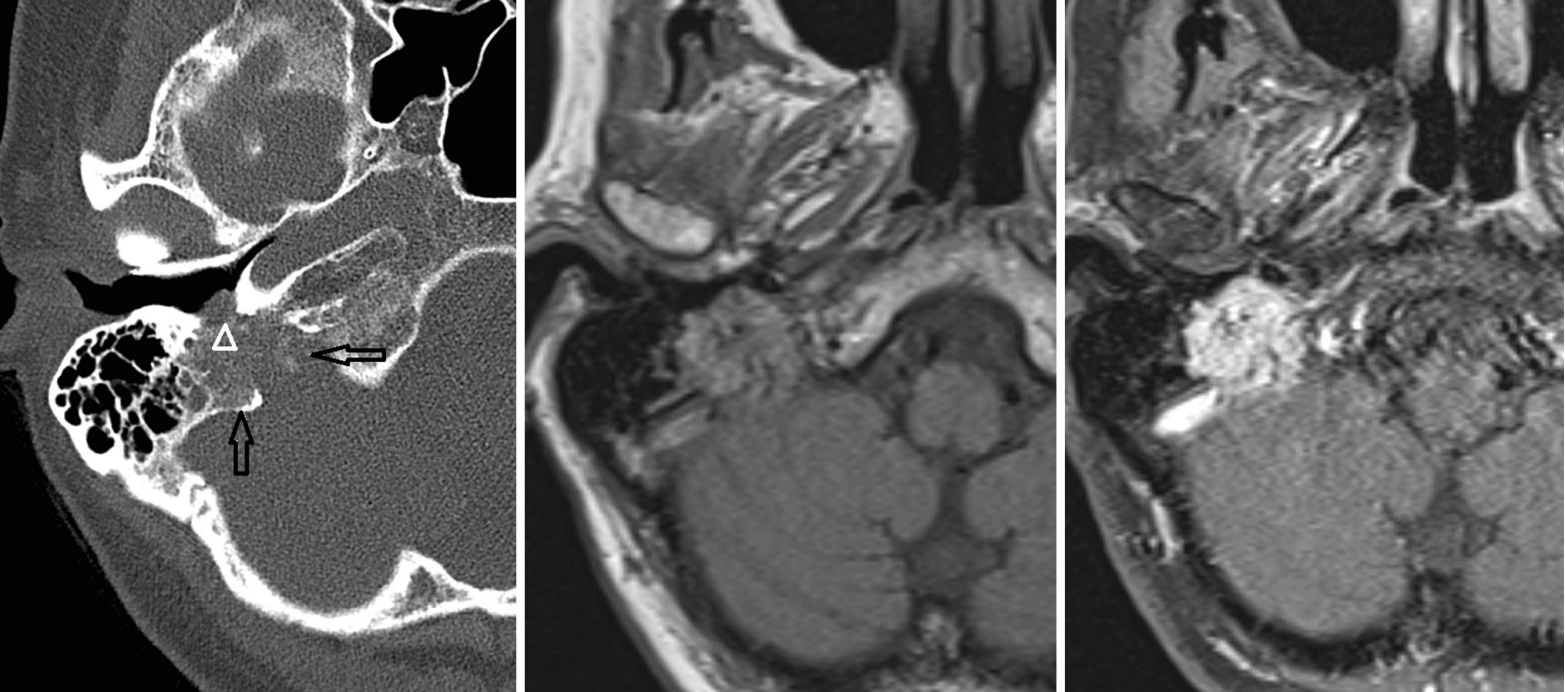
Glomus jugulotympanicum on CT and MRI. *Left* CT demonstrating erosive changes at the jugular bulb (*arrows*). Notice extension of the soft tissue into the middle ear (*arrowhead*). *Middle* Axial T1-W image shows a mixture of signal intensities due to vascular flow voids, which makes up the ‘salt and pepper’ appearance. *Right* Axial contrast-enhanced T1-W with fat suppression demonstrates avid contrast enhancement of the tumor

There is an anatomic close relation between the tympanic cavity and the jugular foramen. As a result, a large percentage of the paragangliomas will present as the jugulotympanic variant due to a tumor located in the jugular foramen with extension through the jugular plate into the hypotympanum. MRI can be used to evaluate bony invasion, although this is usually better appreciated on CT. A CT scan with bone algorithm may show irregular erosions of the adjacent bone (Fig. 5). These bony changes can have a “moth eaten” appearance. The characteristics on CT are being used for the Fisch classification of (jugulo)-tympanic paragangliomas, based on which treatment decisions are made [44].

The classical MRI characteristics of a paraganglioma include a mixture of hypo- and hyper-intensity on T1-W and T2-W sequences, also referred to as a ‘salt-and-pepper’ pattern, based on multiple vascular flow voids within the lesion. Due to the hypervascular nature, a paraganglioma enhances avidly (Fig. 5). Dynamic contrast-enhanced MRA can differentiate a paraganglioma from lymph nodes or other soft tissue lesions, as a paraganglioma will show early contrast enhancement in the arterial contrast phase. In most instances, a hypertrophic ascending pharengeal artery can be identified as the main arterial feeder of the paraganglioma. DSA is not required for diagnosis, but can be performed in case preoperative tumor embolization is considered.

### Osseous Pathology

#### Paget Disease

Paget disease is a primary bone disorder, which can be located in different parts of the skeleton, including the skull and temporal bone. The disease is characterized by osteoclastic resorption, osteoblastic regeneration, and mosaic bone replacement, seen as areas of abnormal lytic or sclerotic changes of bony structures on CT or MRI. Increase in the number and size of local vessels in Paget disease could be the cause of pulsatile tinnitus [45].

#### Otosclerosis

**Fig. 6:**
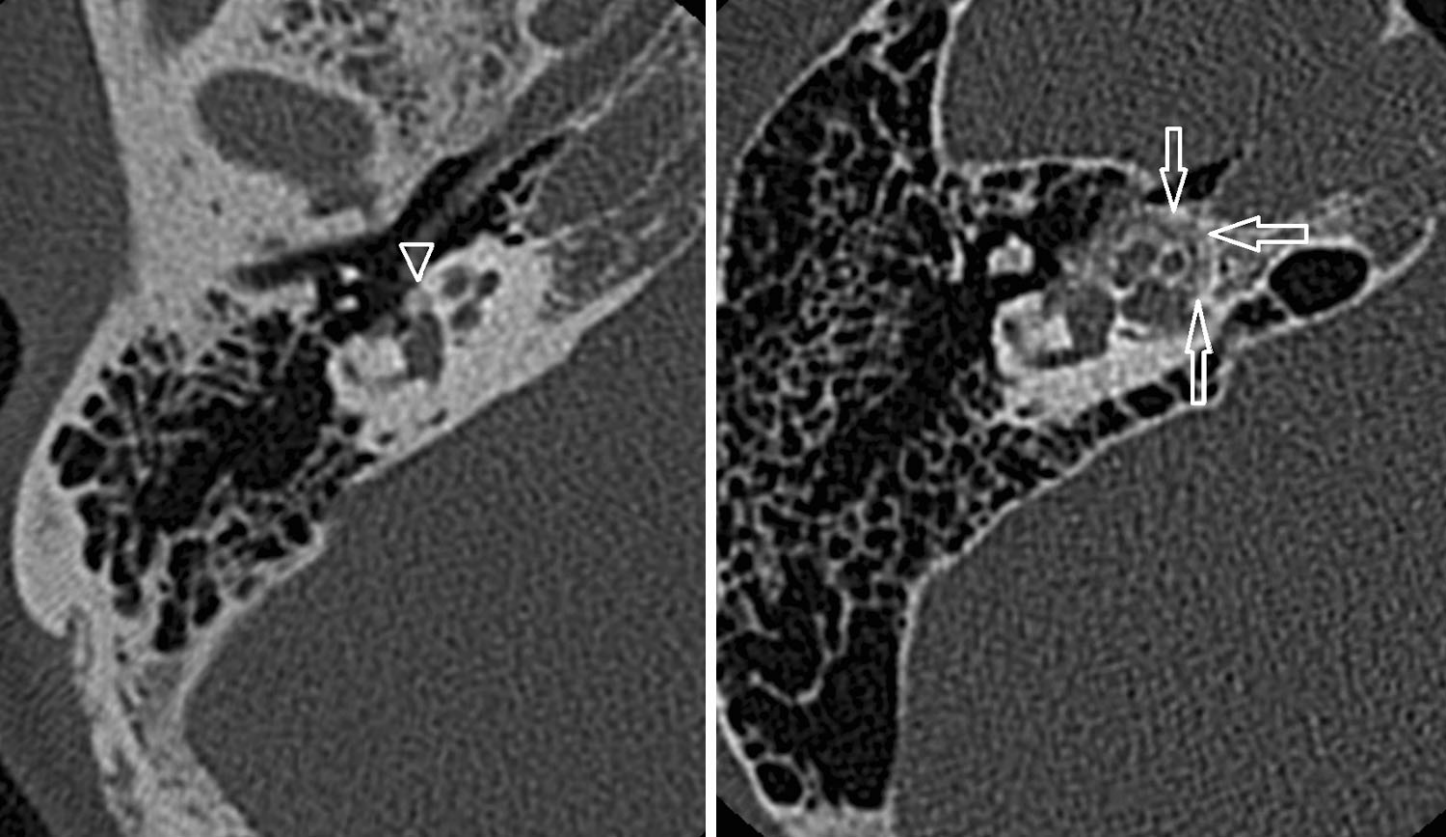
Fenestral (*left*) and cochlear otosclerosis (*right*) on axial thin-sliced CT. Lucency of the fissula antefenestram (*arrowhead*) in fenestral otosclerosis. Lucent halo surrounding the cochlea (*arrows*) in cochlear otosclerosis

Otosclerosis, also known as otospongiosis, is an idiopathic infiltrative process of the petrous bone. It causes both sensorineural and conductive hearing loss, and can be the cause of pulsatile tinnitus [46, 47]. Two types of otosclerosis are differentiated based on the primary region of involvement, fenestral otosclerosis and cochlear otosclerosis. The latter is also referred to as retrofenestral otosclerosis. High-resolution, thin-sliced CT typically shows abnormal hypoattenuated bone in the region of the fissula antefenestram in fenestral otosclerosis (Fig. 6 left). Cochlear otosclerosis appears as a hypoattenuated halo surrounding the cochlea on CT (Fig. 6 right). MRI is considered not sensitive for the diagnosis of otosclerosis, although inhomogeneous high T2 signal, low T1 signal, or subtle contrast enhancement can be seen in the affected petrous bone.

#### Other Osseous Pathology

Highly vascularized bone lesions, like osseous hemangioma, basal meningeoma, Langerhans cell histiocytosis, or bone metastases, have been described as possible causes of pulsatile tinnitus [48–50].

An *osseous hemangioma* is a sharply demarcated expansile intraosseous lesion. Typically, aggravated trabecular thickening is seen on CT with preservation of the inner and outer cortex. MRI will show heterogeneous hyper-intense signal on T1-W and T2-W sequences, and less frequently hypo- or iso-intense signal intensity. A hemangioma enhances diffusely after contrast administration.

**Fig. 7:**
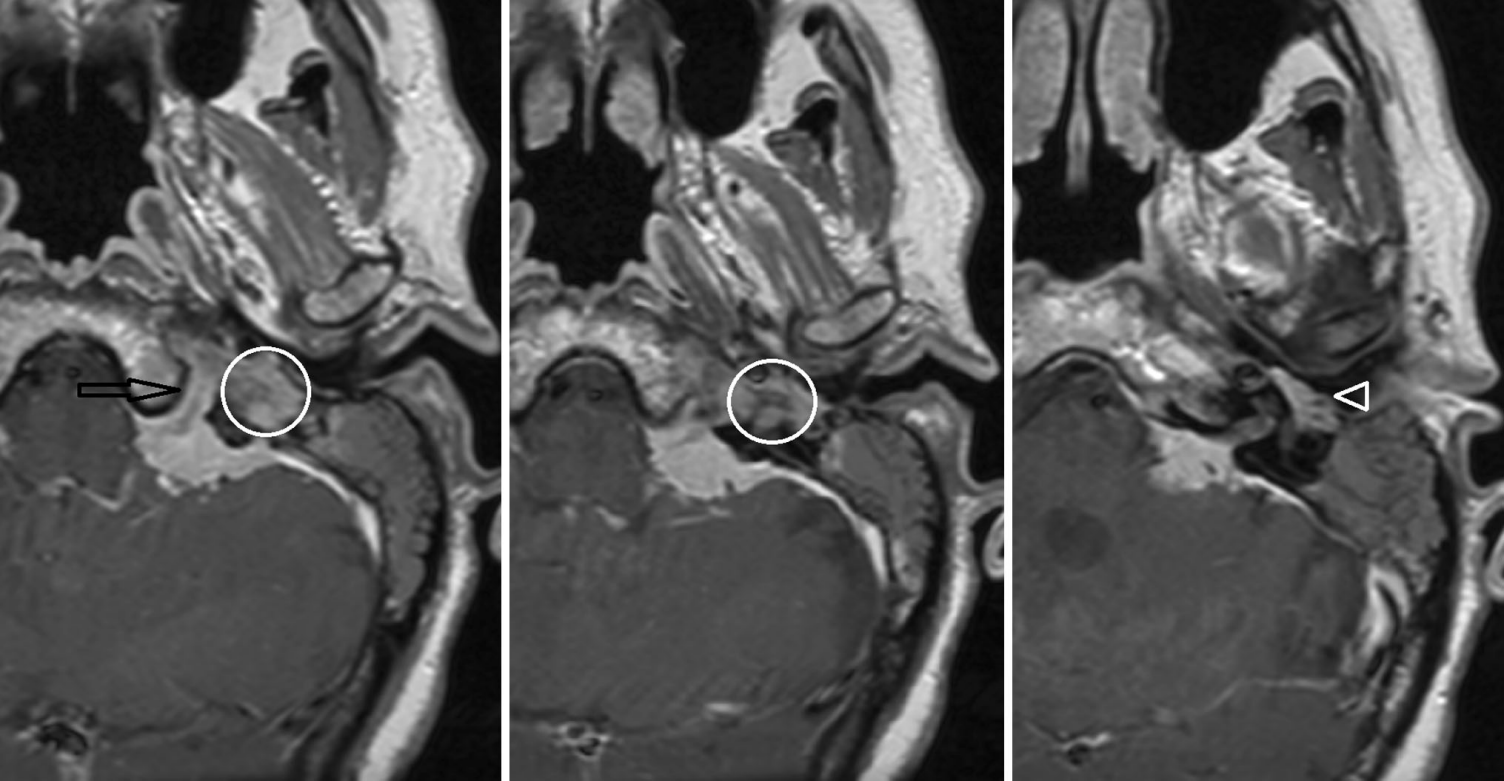
Meningeoma on MRI. Axial contrast-enhanced T1-W images. Enhancing mass located in the left cerebellar-pontine angle with extension into hypoglossal canal (*arrow*), jugular plate (*encircled*), and the middle ear (*arrowhead*)

A *meningioma*, a tumor originating from the meninges, can show permeative changes and sclerosis of the adjacent bone but can also have a significant intraosseous localization. A meningeoma is characterized by homogeneous contrast enhancement and typically shows a dural tail configuration (Fig. 7).

*Langerhans cell histiocytosis* can occur anywhere in the skeleton, and in the head and neck region, the most frequent localizations include the mastoid and the squamous portion of the temporal bone. The affected bone shows lytic, punched-out changes on CT. The affected bone marrow will show signal alterations on MRI.

Examples of typical *highly vascularized bone metastases* include renal cell carcinoma and thyroid carcinoma.

### Idiopathic Intracranial Hypertension

Idiopathic intracranial hypertension (IIH), which predominantly affects young obese women, may cause pulsatile tinnitus, although IIH is primarily characterized by symptoms of headache and blurred vision due to increased cerebrospinal fluid pressure [51••]. Brain MRI typically shows an empty sella and increased cerebrospinal fluid around the optic nerves. The exact pathophysiology of IIH is unknown but can develop in patients with a history of dural sinus trombosis. Dural sinus stenosis or compression can also be observed in IHH. It is therefore advised to perform MRV or CTV in a patient with pulsatile tinnitus and suspicion of IIH.

## Conclusion

Pulsatile tinnitus can lead to significant morbidity, and identification of the underlying cause is important for adequate treatment and prognosis estimation. In general, head and neck MRI and CT provide complementary diagnostic imaging information. Which imaging modality to choose as well as the optimal scanning protocol depends on the estimated a priori chance for finding specific pathology, or the need to rule out more rare but clinical significant disease.

For screening for underlying pathology, and for the evaluation of a possible soft tissue mass or intracranial pathology, evaluation with MRI and MR angiography (MRA) is recommended. In case MRI/MRA does not reveal an underlying cause of pulsatile tinnitus, 4D-CTA can be considered as a non-invasive alternative to DSA for the detection of a dAVF. When pathology of the tympanic cavity or osseous pathology of the temporal bone is suspected based on the clinical evaluation, initial evaluation with CT(A) is advised. The role of DSA is minimized, and can be considered to rule out vascular pathology, in case of high suspicion of vascular pathology while MRI/MRA and CT/(4D-)CTA have not revealed the cause of pulsatile tinnitus. DSA can also be performed for the purpose of treatment planning.

By using an adequate diagnostic imaging strategy, the underlying pathology of pulsatile tinnitus can be identified in the majority of patients.

## Electronic supplementary material

Below is the link to the electronic supplementary material.

Video 1 4D-CTA of dAVF located in the right sigmoid sinus, as shown in Fig. 2 (AVI 11034 kb)

Video 2 DSA of dAVF located in the right sigmoid sinus, as shown in Fig. 2 (AVI 11331 kb)
